# Tai Chi Chuan Alters Brain Functional Network Plasticity and Promotes Cognitive Flexibility

**DOI:** 10.3389/fpsyg.2021.665419

**Published:** 2021-06-29

**Authors:** Lei Cui, Sha Tao, Heng-chan Yin, Qi-qi Shen, Yuan Wang, Li-na Zhu, Xiu-juan Li

**Affiliations:** ^1^College of P.E. and Sports, Beijing Normal University, Beijing, China; ^2^State Key Laboratory of Cognitive Neuroscience and Learning, Beijing Normal University, Beijing, China; ^3^PE Department, Renmin University of China, Beijing, China

**Keywords:** brain plasticity, functional networks, exercise intervention, Tai Chi Chuan, functional magnetic resonance imaging

## Abstract

**Objective:** This study used resting-state functional magnetic resonance imaging to investigate the effects of 8 weeks of Tai Chi Chuan and general aerobic exercise on the topological parameters of brain functional networks, explored the advantages of Tai Chi Chuan for improving functional network plasticity and cognitive flexibility, and examined how changes in topological attributes of brain functional networks relate to cognitive flexibility.

**Methods:** Thirty-six healthy adults were grouped into Tai Chi Chuan (Bafa Wubu of Tai Chi), general aerobic exercise (brisk walking), and control groups. All of the subjects underwent fMRI and behavioral assessment before and after the exercise intervention.

**Results:** Tai Chi Chuan exercise significantly enhanced the clustering coefficient and local efficiency compared with general aerobic exercise. Regarding the nodal properties, Tai Chi Chuan significantly enhanced the nodal clustering coefficient of the bilateral olfactory cortex and left thalamus, significantly reduced the nodal clustering coefficient of the left inferior temporal gyrus, significantly improved the nodal efficiency of the right precuneus and bilateral posterior cingulate gyrus, and significantly improved the nodal local efficiency of the left thalamus and right olfactory cortex. Furthermore, the behavioral performance results demonstrated that cognitive flexibility was enhanced by Tai Chi Chuan. The change in the nodal clustering coefficient in the left thalamus induced by Tai Chi Chuan was a significant predictor of cognitive flexibility.

**Conclusion:** These findings demonstrated that Tai Chi Chuan could promote brain functional specialization. Brain functional specialization enhanced by Tai Chi Chuan exercise was a predictor of greater cognitive flexibility.

## Introduction

Recently, the potential of mind-body exercises for promoting health and preventing diseases has attracted the attention of investigators. Tai Chi Chuan (also known as Taiji, Taijiquan, and Tai Ji Quan) is a popular mind-body exercise and a form of traditional Chinese exercise. Tai Chi Chuan combines the coordination of slow movements with mental focus, deep breathing, and relaxation (Cui et al., 2019). Compared to conventional exercises that usually focus on muscular strength and endurance, Tai Chi Chuan emphasizes mind-body cultivation through slow voluntary movements, full-body stretching and relaxation, diaphragmatic breathing practice, a meditative state of mind and intense mental concentration (Zou et al., 2018). The benefits of Tai Chi Chuan have been widely reported for cognitive function (Lam et al., 2011; Zhang et al., 2018), emotional regulation (Kong et al., 2019), and motor function [e.g., postural control (Ni et al., 2014) and fall prevention (Tousignant et al., 2013)].

To better understand the neural mechanisms underlying these improvements, researchers have used magnetic resonance imaging (MRI). Previous studies have shown that long-term Tai Chi Chuan practice can increase brain volume and cortical thickness (Wei et al., 2013), improve the fractional amplitude of low-frequency fluctuations (fALFF) (Yin et al., 2014; Tao et al., 2017), modulate functional connectivity in older adults (Wei et al., 2017), and change local functional homogeneities (Wei et al., 2014). Our studies have shown that 8 weeks of Tai Chi Chuan intervention has a stronger effect on brain plasticity than general aerobic exercise, which is demonstrated by an increase in gray matter volume and the enhancement of functional connectivity.

The human brain is a formidably complex system in which billions of neurons constitute a dauntingly complicated network (Bullmore and Sporns, 2009; Liao et al., 2017). The characterization of the human brain from a network perspective provides a comprehensive understanding of the structural and functional architectures of the human brain (Liao et al., 2017). Resting-state functional magnetic resonance imaging (rs-fMRI) analyses rely upon spontaneous coupled brain activity, so an increasing number of studies have used rs-fMRI to reveal spontaneous neural activity (Fox and Raichle, 2007). Previous studies have demonstrated that brain networks exhibit small-world attributes characterized by dense local interconnectivity but short characteristic path lengths linking individual network nodes (Stam, 2004; Achard et al., 2006; Bassett and Bullmore, 2006; Achard and Bullmore, 2007; Wang et al., 2009). It supports efficient information segregation and integration with low energy and wiring costs, and several studies suggest that one property of these networks, cost-efficiency, is an important optimization principle (Fornito et al., 2011). Graph theory measures assess the efficiency of information processing within and across intrinsic connectivity brain networks. It helps demonstrate the aspects of the intrinsic topological organization of human brain networks, such as small-worldness, modular organization, and high connectivity (He and Evans, 2010; Craddock et al., 2013). It is a powerful and quantitative approach for examining the segregation and integration of brain networks (Sporns, 2013).

Exploring the small-world architectures is crucially important for understanding the brain mechanisms underlying cognition and behavior (Park and Friston, 2013). However, to date, no research has explored the influence of Tai Chi Chuan on the topological properties of brain networks. The potential role of brain functional networks in the effect of Tai Chi Chuan intervention on cognition remains largely unknown. Besides, whether Tai Chi Chuan, a mind-body exercise that includes mindfulness, has a better effect on cognitive function and brain activity than general aerobic exercise (non-mindfulness) is also worth further discussion. Bafa Wubu of Tai Chi is based on the existing 24-form Tai Chi Chuan, refined and organized systematically from the Bafa Wubu techniques, which are the essence of various types of Tai Chi Chuan (Shaojun Lyu, 2019; Cui et al., 2019). It is a new set of Tai Chi Chuan that is easy to learn and practice and is now being popularized in China.

In summary, we used a resting-state fMRI approach to investigate: (1) whether a long-term Tai Chi Chuan intervention, represented by Bafa Wubu of Tai Chi, could induce changes in the topological properties of functional networks and promote cognitive function and (2) whether this effect is superior to that of general aerobic exercise.

## Materials and Methods

### Participants and Study Design

The 36 healthy adults without regular exercise habits were randomly designated the Tai Chi Chuan intervention group (TCC), general aerobic exercise group (AE), and control group. The three groups (*n* = 12 in each group) were matched with respect to sex. There were no significant differences among the TCC, AE, and control groups in sex, age, handedness, education, or body mass index (BMI; see Table 1).

**Table 1:** Sample characteristics (M ± SD).

**Variables**	**TCC**	**AE**	**Control**	**F**	***p***
Sex (Male/Female)	2/10	2/10	2/10	—	—
Age (years)	21.83 ± 2.48	21.92 ± 2.28	21.75 ± 2.45	0.014	0.986
Handedness (Left/Right)	0/12	0/12	0/12	—	—
Education (years)	16.33 ± 2.23	16.41 ± 2.27	16.33 ± 1.50	0.007	0.993
BMI (kg/m^2^)	20.21 ± 2.54	19.15 ± 2.06	21.71 ± 2.96	3.07	0.06

The experiment used a 3 (group: TCC, AE, control) × 2 (time: pre, post) mixed factorial design with group as the between-subjects factor and time as the within-subjects factor. Subjects were assessed with rs-fMRI scanning and the more-odd shifting task before and after the training period. Each subject first had an 8-min resting-state fMRI scan and then engaged in the more-odd shifting task (only behavioral).

All subjects were right-handed, with no history of psychiatric or neurological disease. This study was approved by the Institutional Review Board of the National Key Laboratory of Cognitive Neuroscience and Learning. All participants provided written informed consent and were paid for their participation. The research was conducted in compliance with the Declaration of Helsinki.

### Exercise Intervention Procedures

The TCC received three weekly sessions of Bafa Wubu of Tai Chi group training for 8 weeks (a total of 24 sessions) in the gym. The three core elements of Tai Chi exercise, namely Xing (body), Qi (breath), and Yi (mind), are commonly referred to as “building Xing,” “conveying Qi,” and “using Yi” in the process of Tai Chi exercise. These three core elements run through the whole Tai Chi Chuan intervention process, but the emphasis varies at different stages. The 1–3 weeks is the stage of “Building Xing,” during which the subject will master all the movements skillfully. During the process, the subject will also be reminded to breathe and try to use mind to guide the movements. The 4–6 weeks are the stage of “Conveying Qi,” which focuses on the respiration of the subject during the intervention. Week 7–8 is mainly a stage of “using Yi,” which emphasizes the integration of mind and finally achieves the harmony of Xing, Qi, and Yi. Each training session consisted of 5 min of warmup, 50 min of continuous sequential practice of learned forms, and 5 min of cool-down. The AE received three weekly sessions of brisk walking group training for 8 weeks in the same gym. Each training session consisted of 5 min of warmup, 50 min of continuous sequential practice, and 5 min of cool-down. Details of the interventions were introduced in our previous paper (Cui et al., 2019). The control group was instructed to maintain their original daily routines and physical activity habits and not to engage in any new or additional exercise interventions.

Using a Polar watch (Polar Electro Oy, Kempele, Finland) to monitor the participants' heart rates during the exercise sessions, we found that the intensity of the 50-min continuous Tai Chi Chuan practice stimulated the heart rates of the participants to reach ~67.995 ± 1.385% (range = 66.61–69.38%) of the individuals' age-predicted maximal heart rates (HRmax) on average, and thus the Tai Chi Chuan intervention could be considered moderate intensity (60–69% HRmax) endurance exercise according to the classification of the American College of Sports Medicine (Wu et al., 2018). The heart rate under general aerobic practice reached ~68.28 ± 1.64% (range = 66.64–69.92%) of the individual participants' age-predicted maximal heart rate (HRmax) on average.

### MRI Data Acquisition

For each participant, we applied rs-fMRI scanning (8 min) during both the pre- and post-intervention periods. Images were acquired on a 3.0 T MRI system (Siemens Magnetom Prisma; Erlangen, Germany) with a 64-channel head coil in Beijing Normal University Imaging Center for Brain Research.

Functional images were obtained using an echo-planar sequence sensitive to blood oxygenation level-dependent contrast (Xu et al., 2016; Cui et al., 2019): TR = 2,000 ms, TE = 30 ms; FA = 90°, slice thickness = 3.5 mm, 33 axial slices, voxel size=3.5 × 3.5 × 3.5 mm, FOV = 224 × 224 mm, 240 volumes. The participants were instructed to keep their eyes open (not fixated condition) without falling asleep and to move as little as possible (Xu et al., 2014; Zeng et al., 2014; Soares et al., 2016). As assessed by a questionnaire, no subjects reported falling asleep during the scanning or being uncomfortable during or after the procedure. In addition, a high resolution three-dimensional T1-weighted magnetization-prepared rapid gradient-echo images were acquired (Xu et al., 2016; Cui et al., 2019): TR = 2,530 ms, TE = 2.98 ms, inversion time = 1,100 ms, FA = 7°, slice thickness = 1 mm, 192 sagittal slices, voxel size = 0.5 × 0.5 × 1 mm, FOV = 224 × 256 mm.

### MRI Data Analysis

Imaging data preprocessing, correlation matrix and graph construction, and functional networks analysis was implemented using GRETNA2.0.0 (https://www.nitrc.org/projects/gretna), which is based on Statistical Parametric Mapping 12 (SPM 12: http://www.fil.ion.ucl.ac.uk/spm), available on the MATLAB R2013b platform.

#### Resting-State fMRI Image Preprocessing

The following conventional steps were accomplished (Xu et al., 2016; Cui et al., 2019): (1) discarding the first 10 time points, allowing for signal equilibrium and adaptation of the participants to the scanning noise, (2) compensation of systematic slice-dependent time shifts, (3) correction for head movement with rigid body translation and rotation parameters, no dataset was excluded according to the criteria of spatial movement in any direction <2 mm translation or 2° rotation, (4) warping individual functional images to the standard MNI space by applying the transformation matrix that can be derived by registering the T1 image (co registered with functional images) into the MN I template by using unified segmentation, and then resampled to 3-mm cubic voxels, (5) spatial smoothing with a 4-mm full-width at half-maximum Gaussian kernel, (6) removing the linear trend of signal with time, (7) bandpass (0.01–0.08 Hz) filtering to decrease physiological noise, and (8) regression of nuisance variables including head motion parameters (Friston-24), the white matter signal averaged from the deep cerebral white matter (WM) and the cerebrospinal fluid (CSF) signal averaged from the ventricles to further reduce non-neuronal contributions (Friston et al., 1996).

#### Correlation Matrix and Graph Construction

The processing steps were as follows: (1) The AAL90 atlas (Tzourio-Mazoyer et al., 2002) was used to divide the brain in each individual space to define 90 network nodes. Each node corresponded to a brain region, and the time series of all voxels in each node was extracted. (2) The network's edges were defined by calculating Pearson's correlation coefficients between the time series of 90 nodes to obtain the 90 × 90 functional connectivity matrix of each individual space and then converting the functional connectivity matrix into the *Z*-value matrix by Fisher's Z transform so that the data were closer to the normal distribution. (3) In this study, we chose the sparsity, S, to define threshold measurements for the between-group comparisons of the small-world parameters. The threshold range of sparsity was identified as 0.05–0.5(Watts and Strogatz, 1998; He et al., 2007; Sporns, 2013) in increments of 0.02.

#### Brain Functional Networks Analysis

The brain functional network was analyzed by using graph theoretical approaches. The following global network measurements were calculated using different thresholds: small-worldness (σ), clustering coefficient (C_*p*_), characteristic path length (L_*p*_), global efficiency (E_*g*_), and local efficiency (E_*loc*_). For regional nodal characteristics, we considered the nodal clustering coefficient (NC_*p*_), nodal efficiency (N_*e*_), and nodal local efficiency (NL_*e*_) of each node. We calculated the integrated area under the curve (AUC) for each network measure, providing a summarized scalar for topological characterization of brain networks independent of the single threshold selection (He et al., 2007). All topological parameters of the brain functional networks calculated in this study were presented in Table 2 (Bullmore and Sporns, 2009; Wang et al., 2009; Rubinov and Sporns, 2010).

**Table 2:** Topological parameters of the brain functional networks.

**Network properties**	**Characters**	**Descriptions**
Global properties	C*_*p*_*	Clustering coefficient of the network. It measures the extent of local cluster or cliquishness of the network and is the most commonly used measure of functional segregation.
	L*_*p*_*	Characteristic path length of the network. It is the average shortest path length between all pairs of nodes in the network and is the most commonly used measure of functional integration.
	E*_*g*_*	Global efficiency of the network. It measures the extent of information propagation through the whole network and is the most commonly used measure of functional integration.
	E*_*loc*_*	Local efficiency of the network. It measures the mean local efficiency of the network and is the most commonly used measure of functional segregation.
	σ	Small-worldness of the network. Small-world networks, reflects an optimal balance of functional integration and segregation, often fulfill the σ > 1.
Nodal properties	NC*_*p*_*	Nodal clustering coefficient. It measures the extent of interconnectivity among the neighbors of the node and is the most commonly used measure of functional segregation.
	N*_*e*_*	Nodal global efficiency. It measures the extent of information transmission of the node with all other nodes in the network and is the most commonly used measure of functional integration.
	NL*_*e*_*	Nodal local efficiency. It measures the extent of information transmission among the neighbors of the node and is the most commonly used measure of functional segregation.

For intervention effects in global network measures, comparisons were performed among three groups (TCC, AE, and control) using repeated measures analysis of variance (ANOVA) followed by one-way ANOVA with Bonferroni *post-hoc* testing in SPSS 25.0. For intervention effects of regional nodal characteristics, the property change values (postexercise intervention minus pre-exercise intervention) at 90 nodes for each participant were extracted, and one-way ANOVA was performed in GRETNA to determine the brain regions with significant changes in node properties, followed by *post-hoc* statistical analysis in SPSS 25.0. The results of the brain network analyses were visualized using the BrainNet Viewer software package (http://www.nitrc.org/project/bnv/).

### Cognitive Flexibility Assessments and Analysis

**Figure 1:**
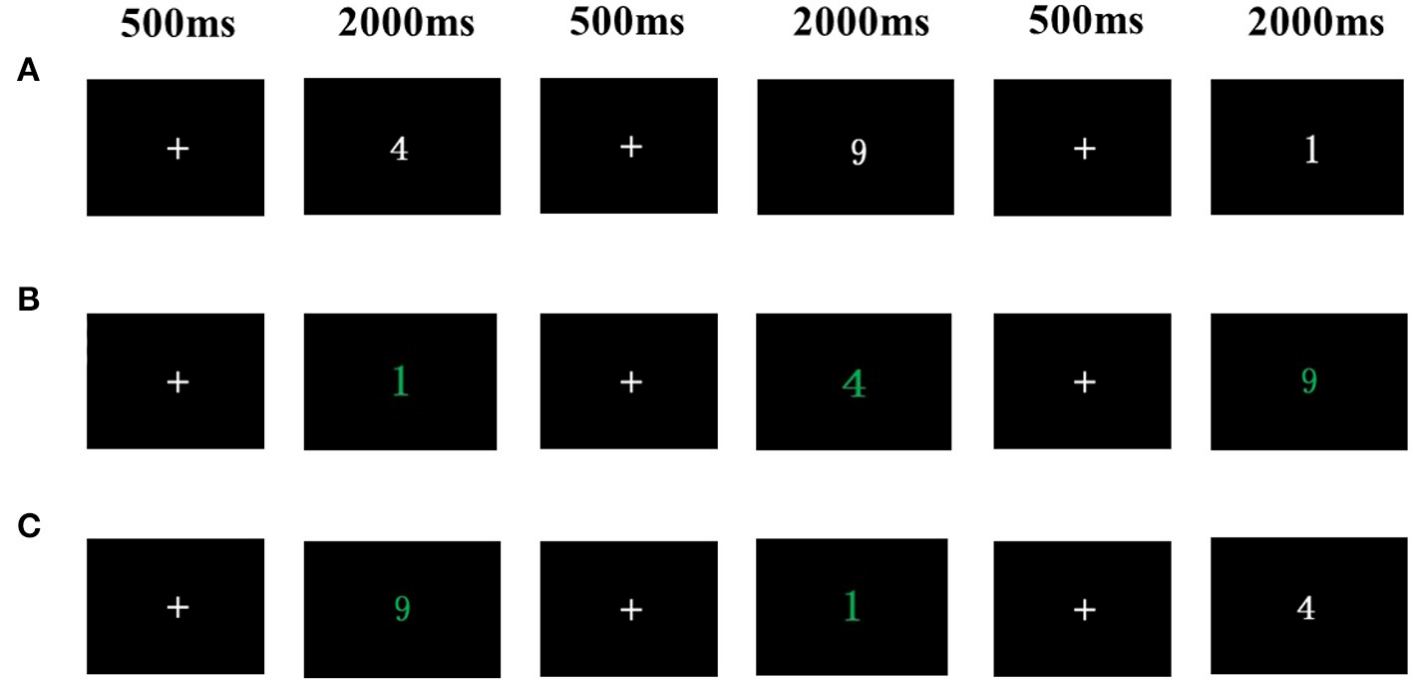
More-odd shifting task. **(A)** Condition1: Participants determined whether the white digit presented was greater or less than 5. **(B)** Condition 2: Participants determined whether the green digit presented was odd or even. **(C)** Condition 3: A white or green digit was presented at random and participants responded accordingly.

The more-odd shifting task (Hillman et al., 2006; Chen et al., 2014; Huang et al., 2015) was used to assess cognitive flexibility (Figure 1). The task consists of three conditions. Condition 1 involved 16 homogeneous trials in which the digits were printed in white. Participants determined whether the white digit presented was greater or less than 5. Condition 2 involved 16 homogeneous trials in which the digits were printed in green. Participants determined whether the green digit presented was odd or even. Condition 3 involved 32 heterogeneous trials that included both white and green digits (16 trials each). White or green digits were presented at random and participants responded accordingly. The cognitive flexibility was calculated as the reaction time difference between the heterogeneous (the average of the condition 3) and homogeneous (the average of the condition 1 and 2) conditions. The task presentation and data collection were performed using E-Prime software 2.0 (Psychology Software Tools Inc., Pittsburgh, USA) on the computer.

Statistical analysis was performed in SPSS 25.0 software (SPSS Inc., Chicago, IL, USA). We compared results before and after the exercise intervention with 3-group× 2-time Repeated Measures Factorial ANOVA, followed by Bonferroni *post-hoc* testing. Pearson correlation was used to estimate the relationship between changes in topological parameters of brain functional networks and changes in cognitive flexibility with Tai Chi Chuan training. A stepwise regression was undertaken to identify the statistically significant predictors.

## Results

### Global Network Properties

Network analysis showed that all three groups satisfied small-world topology (σ > 1) before and after exercise intervention over a wide range of sparsity (0.05 ≤ *S* ≤ 0.5; Wang et al., 2009).

**Figure 2:**
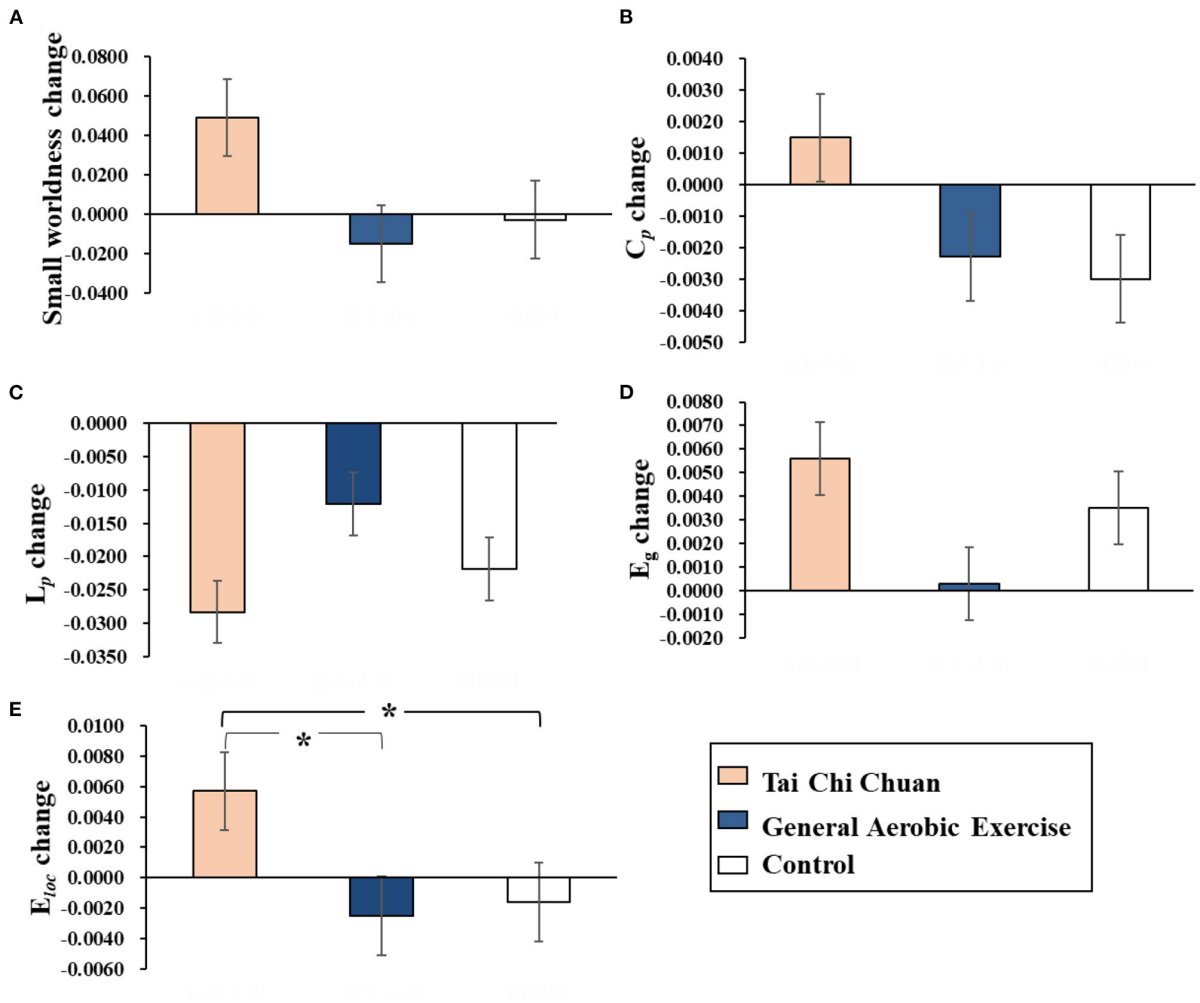
Results of Bonferroni *post-hoc* statistical analysis of global network property changes (post minus pre). ^*^p < 0.05. **(A)** Small-worldness. **(B)** C_*p*_. **(C)** L_*p*_. **(D)** E_*g*_. **(E)** E_*loc*_. C_*p*_, clustering coefficient; L_*p*_, characteristic path length; E_*g*_, global efficiency; E_*loc*_, local efficiency.

A 3-group× 2-time Repeated Measures Factorial ANOVA revealed that there were no significant group, time, or interaction effects on small-worldness, C_*p*_, L_*p*_, or E_*g*_. Moreover, the interaction between group and time was significant for E_*loc*_ (*Wilks'* λ = 0.811, *F*_(1, 33)_ = 3.837, *p* = 0.032 < 0.05, *partial* η^2^ = 0.189). E_*loc*_ changes (post minus pre) were compared before and after the 8 weeks of exercise intervention among the three groups. The results showed that E_*loc*_ was significantly elevated in the TCC group compared to the control group (*p* = 0.031< 0.05, *Cohen's d* = 0.911) and AE group (*p* = 0.016 < 0.05, *Cohen's d* = 0.979). There was no significant difference between the AE group and the control group (*p* = 0.781> 0.05, *Cohen's d* = 0.123; Figure 2).

### Nodal Properties

#### Nodal Clustering Coefficient

NC_*p*_ among the three groups was detected in the left thalamus (THA. L; cluster size: 313; peak MNI coordinates: −10.85, −17.56, 7.98; *F* = 4.520, *p* = 0.018 < 0.05, *partial* η^2^ =0.215), right olfactory cortex (OLF.R; cluster size:81; peak MNI coordinates: 10.43, 15.91, −11.26; *F* = 4.013, *p* = 0.028 < 0.05, *partial* η^2^ =0.196) and left inferior temporal gyrus (ITG. L; cluster size: 941; peak MNI coordinates: −49.77, −28.05, −23.17; *F* = 3.550, *p* = 0.042 < 0.05, *partial* η^2^ =0.175). Marginal significance was observed for the left olfactory cortex (OLF. L; cluster size: 87; peak MNI coordinates: −8.06, 15.05, −11.46; *F* = 3.133, *p* = 0.057 < 0.06, *partial* η^2^ =0.160).

**Figure 3:**
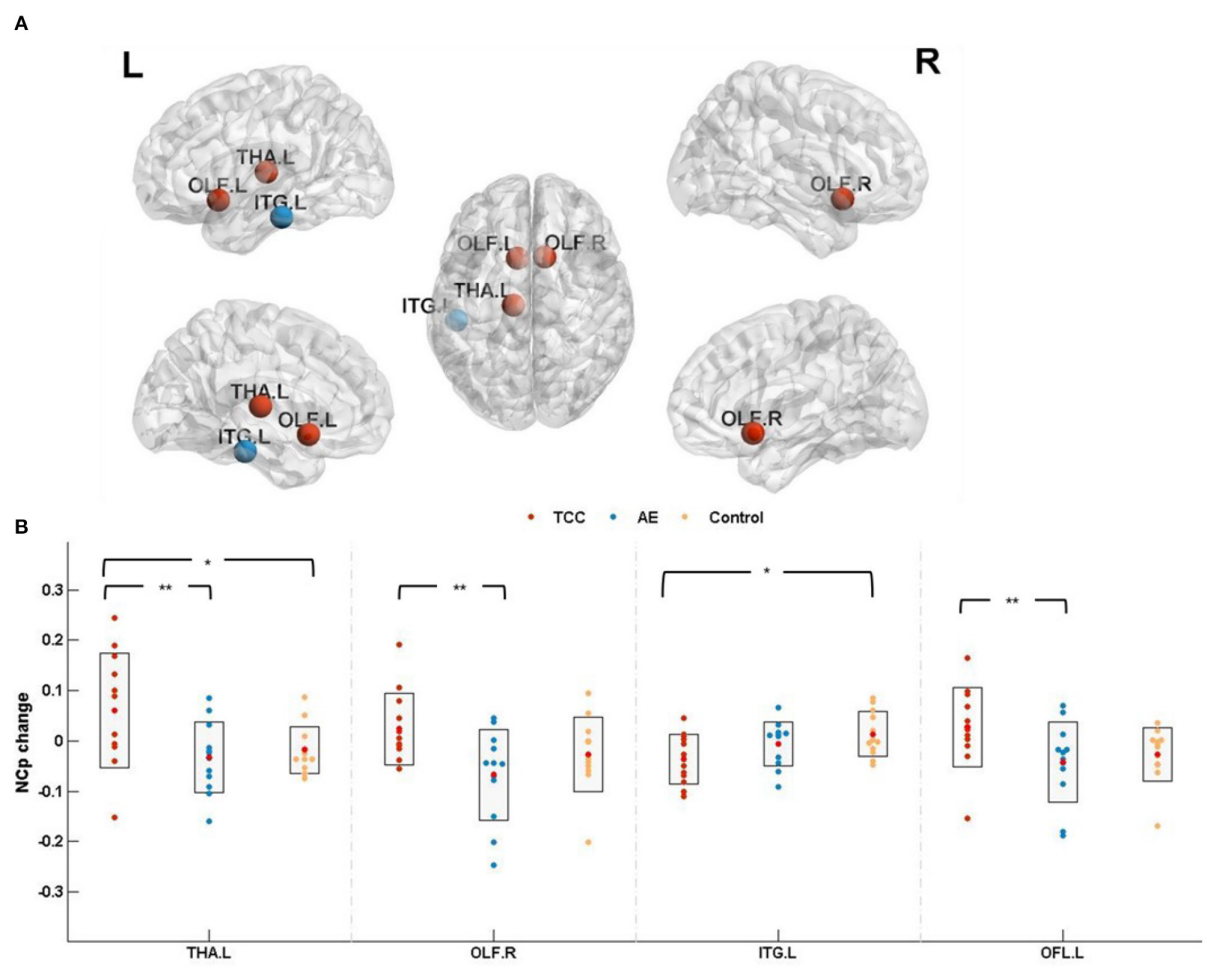
Nodal clustering coefficient results. **(A)** The results of nodal clustering coefficient changes are presented on axial slices of the gray matter templates (MNI), Bonferroni-corrected. **(B)** The results of *post-hoc* statistical analysis of NC_*p*_ changes (post minus pre). +indicates *p* < 0.06 (marginal significance), ^*^p < 0.05, ^**^p < 0.01. THA.L, left thalamus; OLF.R, right olfactory cortex; ITG.L, left inferior temporal gyrus; OFL.L, left olfactory cortex; NC_*p*_, nodal clustering coefficient.

NC_*p*_ changes (post minus pre) were compared before and after the 8 weeks of exercise intervention among the three groups. The results showed that NC_*p*_ was significantly elevated in the THA. L (*p* = 0.025 < 0.05, *Cohen's d* = 0.899) and significantly reduced in the ITG. L (*p* = 0.013<0.05, *Cohen's d* = 1.047) in the TCC group compared to the control group. Compared with the AE group, the TCC group was associated with a significant NC_*p*_ increase in the THA. L (*p* = 0.008 < 0.01, *Cohen's d* = 0.991), OFL. R (*p* = 0.008 < 0.01, *Cohen's d* = 1.117), and OFL. L (*p* = 0.023 < 0.05, *Cohen's d* = 0.796). No significant differences were observed between the AE and control groups (Figure 3).

#### Nodal Efficiency

N_*e*_ among the three groups was detected in the right posterior cingulate (PCG. R; cluster size: 87; peak MNI coordinates: 7.44, −41.81, 21.87; *F* = 6.027, *p* = 0.0059 < 0.01, *partial* η^2^ =0.268), left posterior cingulate (PCG. L; cluster size:137; peak MNI coordinates: −4.85, −42.92, 24.67; *F* = 5.362, *p* = 0.0096 < 0.01, *partial* η^2^ =0.245), and right cuneus (CUN. R; cluster size: 434; peak MNI coordinates: 13.51, −79.36, 28.23; *F* =3.346, *p*=0.048 < 0.05, *partial* η^2^ =0.169). Marginal significance was observed for the left hippocampus (HIP. L; cluster size: 273; peak MNI coordinates: −25.03, −20.74, −10.13; *F* = 3.130, *p* = 0.057 < 0.06, *partial* η^2^ =0.159) and right precuneus (PCUN. R; cluster size: 935; peak MNI coordinates: 9.98, −56.05, −43.77; *F* =3.262, *p* = 0.051 < 0.06, *partial* η^2^ = 0.165).

**Figure 4:**
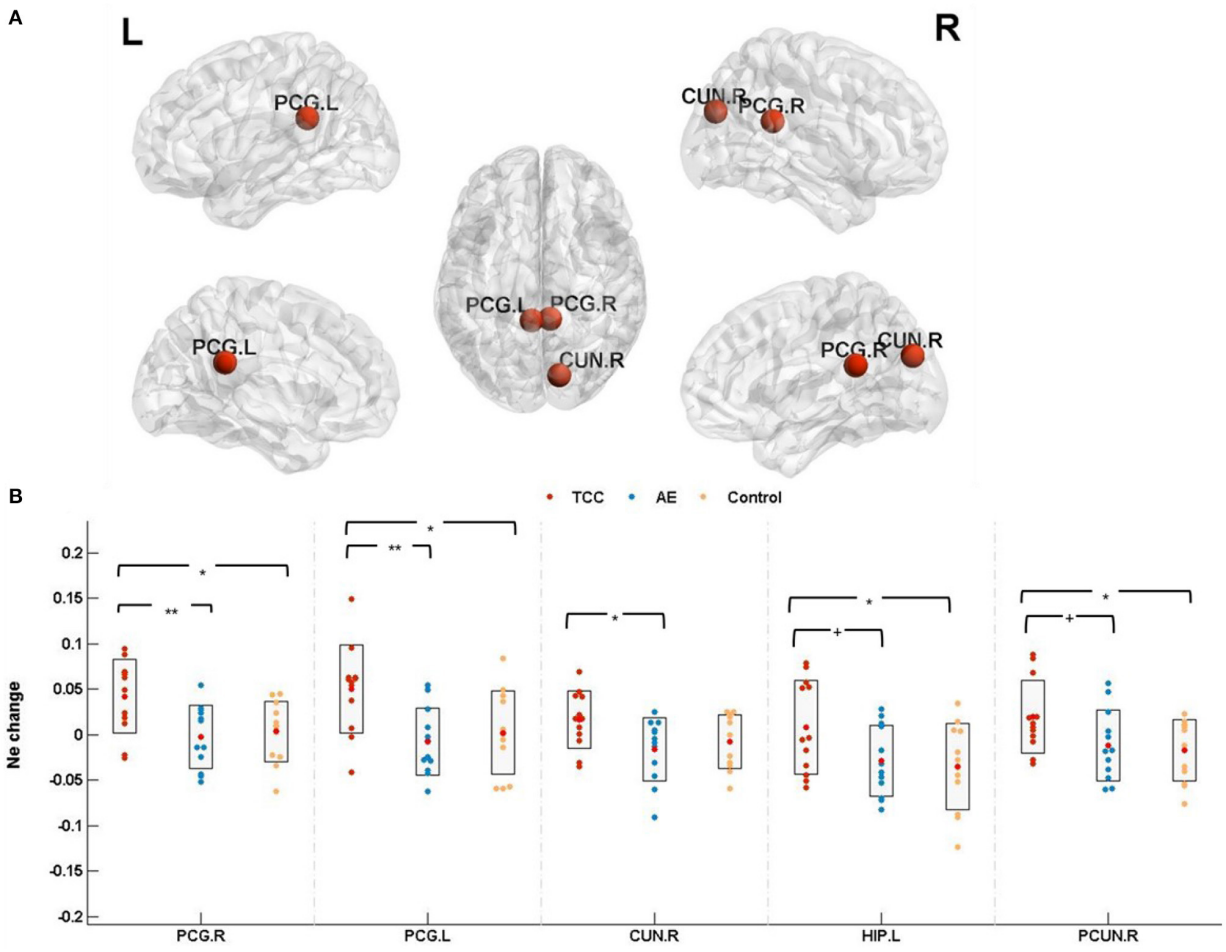
Nodal efficiency results. **(A)** The results for nodal efficiency changes are presented on axial slices of the gray matter templates (MNI), Bonferroni-corrected. **(B)** The results of *post-hoc* statistical analysis of N_*e*_ changes (post minus pre). ^+^*p* < 0.06 (marginal significance); ^*^p < 0.05; ^**^p < 0.01. PCG.R, right posterior cingulate; PCG.L, left posterior cingulate; CUN.R, right cuneus; HIP.L, left hippocampus; PCUN.R, right precuneus; N_*e*_, nodal efficiency.

N_*e*_ changes (post minus pre) were compared before and after the 8 weeks of exercise intervention among the three groups. The results showed that N_*e*_ was significantly elevated in the PCG. R (*p* = 0.011 < 0.05, *Cohen's d* = 1.027), PCG. L (*p* = 0.013 < 0.05, *Cohen's d* = 1.054), and HIP. L (*p* = 0.024 < 0.05, *Cohen's d* = 0.885) in the TCC group compared to the control group. Compared with the AE group, the TCC group was associated with a significant N_*e*_ increase in the PCG. R (*p* = 0.008 < 0.01, *Cohen's d* = 1.354), PCG. L (*p* = 0.008 < 0.01, *Cohen's d* = 1.181), and CUN. R (*p* = 0.023 < 0.05, *Cohen's d* = 0.981) and a marginally significant N_*e*_ increase in the HIP. L (*p* = 0.057 < 0.06, *Cohen's d* = 0.815) and PCUN. R (*p* = 0.052 < 0.06, *Cohen's d* = 0.784). No significant differences were observed in the AE or control groups (Figure 4).

#### Nodal Local Efficiency

NL_*e*_ among the three groups was detected in the OLF. R (cluster size: 81; peak MNI coordinates: 10.43, 15.91, −11.26; *F* = 4.456, *p* = 0.019 < 0.05, *partial* η^2^ = 0.155) and THA. L (cluster size: 313; peak MNI coordinates: −10.85, −17.56, 7.98; *F* = 4.209, *p* = 0.0235 < 0.05, *partial* η^2^ = 0.203).

**Figure 5:**
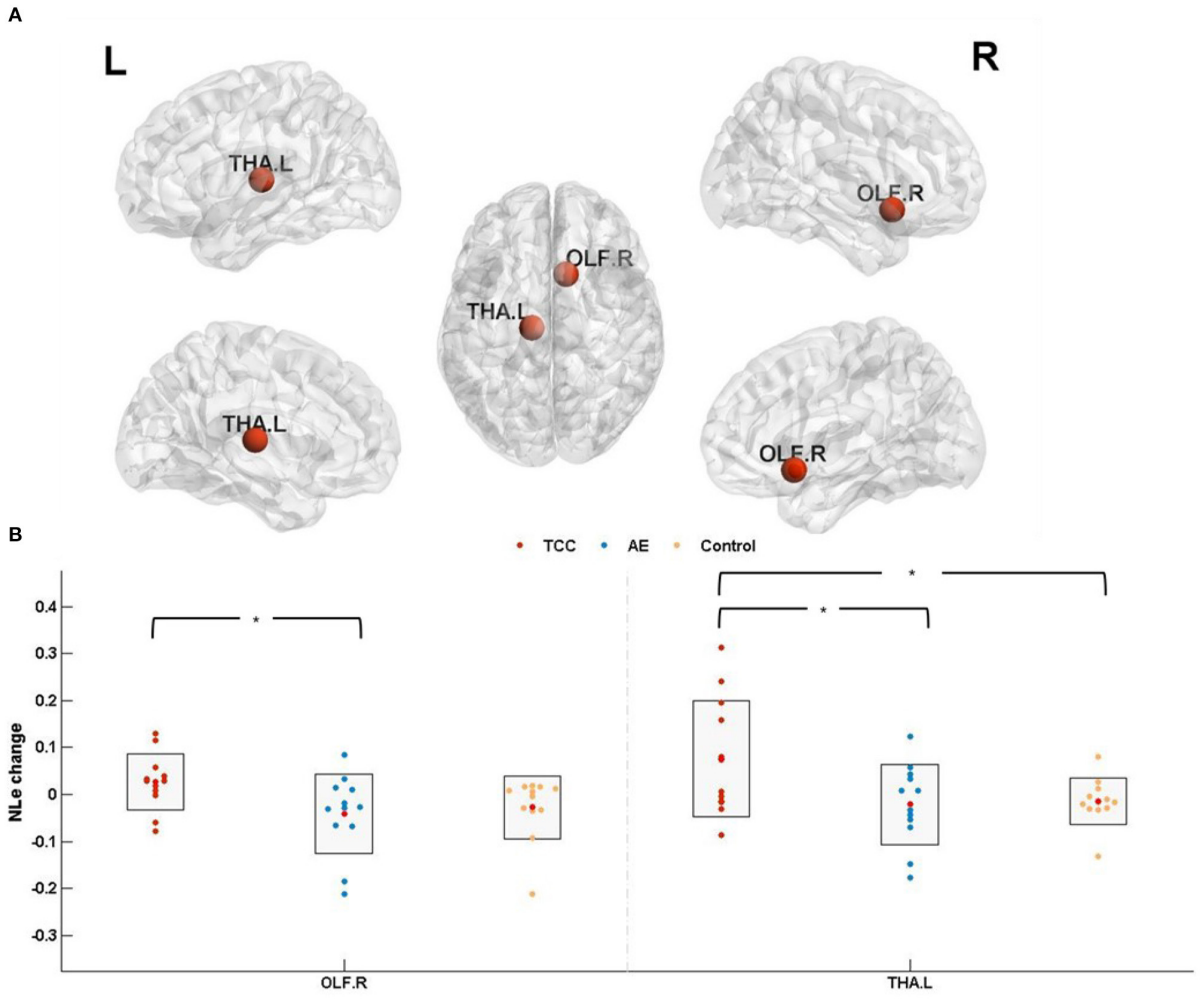
Nodal local efficiency results. **(A)** The results of nodal efficiency changes are presented on axial slices of the gray matter templates (MNI); Bonferroni-corrected. **(B)** The results of *post-hoc* statistical analysis of NL_*e*_ changes (post minus pre). ^*^p < 0.05; ^**^p < 0.01. OLF.R, right olfactory cortex; THA.L, left thalamus; NL_*e*_, nodal local efficiency.

NL_*e*_ changes (post minus pre) were compared before and after the 8 weeks of exercise intervention among the three groups. The results showed that NL_*e*_ was significantly elevated in the THA. L (*p* = 0.021 < 0.05, *Cohen's d* = 0.957) in the TCC group compared to the control group. Compared with the AE group, the TCC group was associated with a significant NL_*e*_ increase in the OLF. R (*p* = 0.014 < 0.05, *Cohen's d* = 1.104) and THA. L (*p* = 0.026 < 0.05, *Cohen's d* = 0.910). No significant differences were observed between the AE and control groups (Figure 5).

### Cognitive Flexibility

A 3-group× 2-time Repeated Measures Factorial ANOVA showed that there were no significant group or time main effects on cognitive flexibility performance. The interaction between group and time had a significant effect [*Wilks'* λ=0.772, *F*_(1, 33)_ = 4.863, *p* = 0.014 < 0.05, *partial* η^2^ = 0.228]. Cognitive flexibility changes (post minus pre) were compared before and after the 8 weeks of exercise intervention among the three groups. The results showed that cognitive flexibility was significantly increased in the TCC group compared to the control group (*p* = 0.012 < 0.05, *Cohen's d* = 1.179). There was no significant difference between the AE group and the control group (*p* = 0.227 > 0.05, *Cohen's d* = 0.456).

### Correlations Between Topological Parameters of Brain Functional Networks and Cognitive Flexibility

We found a significant correlation between NC_*p*_, which was elevated in THA.L and better after the Tai Chi Chuan intervention, and cognitive flexibility performance (*r* = 0.680, *p* = 0.015 < 0.05; Table 3). Additionally, Tai Chi Chuan induced NC_*p*_ changes in THA.L was a significant predictor of cognitive flexibility (*R*^2^ = 0.462, *p* = 0.015 < 0.05; Table 4).

**Table 3:** The results of Pearson correlation between topological parameters changes and cognitive flexibility changes.

		**E*_***loc***_***	**N*_***e***_*-PCG.L**	**N*_***e***_*-PCG.R**	**N*_***e***_*-CUN.R**	**N*_***e***_*-HIP.L**	**N*_***e***_*-PCUN.R**	**NL*_***e***_*-OLF.R**	**NL*_***e***_*-THA.L**	**NC*_***p***_*-THA.L**	**NC*_***p***_*-OFL.R**	**NC*_***p***_*-ITG.L**	**NC*_***p***_*-OFL.L**
Cognitive flexibility	*r*	−0.19	0.10	−0.14	−0.21	−0.36	−0.40	−0.17	−0.54	−0.68^*^	−0.31	0.55	−0.32
	*p*	0.55	0.76	0.67	0.51	0.25	0.20	0.60	0.07	0.02	0.33	0.07	0.32

* *p < 0.05*.

**Table 4:** Results of stepwise regression of topological parameters changes with cognitive flexibility changes.

**Model**	***Beta* coefficients**	***R^**2**^***	**Adjusted *R^**2**^***	***F***	***p***
NCp-THA.L	−0.680	0.462	0.408	8.589^*^	0.015

* *p < 0.05*.

## Discussion

To the best of our knowledge, this study is the first exercise intervention study to employ whole-brain network analysis and compare the impact of Tai Chi Chuan and general aerobic exercise on brain functional network specialization and efficiency. Compared with general aerobic exercise, 8 weeks of Tai Chi Chuan significantly enhanced the local efficiency, significantly enhanced the nodal clustering coefficient of the bilateral olfactory cortex and left thalamus, significantly reduced the nodal clustering coefficient in the left inferior temporal gyrus, significantly improved the nodal efficiency in the right precuneus and bilateral posterior cingulate, and significantly improved the nodal local efficiency in the left thalamus and right olfactory cortex. Moreover, the Tai Chi Chuan induced nodal clustering coefficient change in the left thalamus was a significant predictor of cognitive flexibility.

### Brain Functional Network Specialization and Efficiency Enhanced With Tai Chi Chuan Intervention

A Tai Chi Chuan intervention can indeed change brain functional plasticity. This conclusion agrees with the findings of prior studies. Several studies have also found that Tai chi Chuan intervention can change brain function in older adults (Chang et al., 2014; Li et al., 2014). Brain functional network attributes can be summarized as functional segregation and functional integration. Functional segregation in the brain is the ability for specialized processing to occur within densely interconnected groups of brain regions (Rubinov and Sporns, 2010). We found that the Tai Chi Chuan intervention could enhance local efficiency, which is an indicator of functional separation and reflects the information transmission efficiency between a network node and its neighbors, namely, the capability of local brain information transmission and processing. A previous cross-sectional design study investigated the differences in functional specialization (by measuring voxel-mirrored homotopic connectivity [VMHC]) between TCC practitioners and healthy controls. These researchers observed reduced VMHC values in the middle frontal gyrus in Tai Chi Chuan practitioners and significant negative relationships between total Tai Chi Chuan practice time and VMHC in the precuneus and precentral gyrus (Chen et al., 2020). These findings indicate that Tai Chi Chuan practice could enhance functional specialization. Our study found that an 8-week Tai Chi Chuan exercise intervention could improve the local efficiency, indicating that Tai Chi Chuan could improve the ability for global brain functional separation, enhance the efficiency of local information processing and transmission of brain networks, and make the brain function more specialized.

Changes in node attributes also confirm the above finding. As a functional separation indicator, the higher clustering coefficient is, the more efficient the brain network is in processing information. The node clustering coefficient indicates how well a brain region connects with its adjacent region, suggesting that the modular processing ability between the brain region and the adjacent brain region is enhanced. A study has shown that the functional connectivity of the brain during a simple motor learning task (three training sessions in 5 days) is inhomogeneous and is segregated into communities that can each perform unique functions (Bassett et al., 2011). In this study, each Tai Chi Chuan practice includes a lot of processes of movement learning and control. Through continuous intensive practice, the modularization of the brain is enhanced, which is reflected in the enhancement of clustering coefficients in some brain regions.

The enhanced nodal clustering coefficient in the bilateral olfactory cortex and left thalamus suggests that the three brain regions are more closely related to their neighbors. At the same time, the nodal local efficiency of the left thalamus and right olfactory cortex was improved, indicating enhanced modularization between these two regions and the adjacent regions, and increased transmission efficiency. As a crucial crossroads in the brain, the thalamus may play important roles in sensory perception, attention, sleep, arousal, working memory, behavioral flexibility, goal-directed behavior, and rewarding responses (Mitchell et al., 2014; Courtiol and Wilson, 2015). Recent studies have found that elderly people who practice Tai Chi for a long time have a larger volume of gray matter in the thalamus, and the volume of gray matter in the thalamus is positively correlated with meditation levels and emotional stability (Liu et al., 2019). A review of Tai Chi and cognition in the elderly have found that Tai Chi may improve the brain and cognition by promoting a meditative state (Chang et al., 2014). Tai Chi Chuan is a mindfulness exercise that includes lots of meditative elements. The improvement of the level of mindfulness or meditation may be the reason why Tai Chi Chuan enhances the thalamic clustering coefficient in this study. For patients with mild cognitive impairment (MCI) and Alzheimer's disease (AD), the entorhinal cortex is vulnerable (Devanand et al., 2008; Lazarov and Marr, 2010). Odor identification ability is impaired in patients with AD (Baek et al., 2020). In our study, the increased nodal clustering coefficient in the bilateral olfactory cortex indicated that the connections between the olfactory cortex and the adjacent brain regions were enhanced. Qi (breath) and Yi (mind) also are the core elements of Tai Chi exercise. Tai Chi Chuan exercise requires quieting the mind while concentrating on slow and gentle movements. The mindful breath and awareness meditation incorporate attention to somatosensory experiences, thereby promoting the improvement of sensory and perceptual abilities.

Nodal global efficiency measures the degree and efficiency of information transmission within the network. This study also found that N_*e*_ was improved in the right precuneus, bilateral right posterior cingulate, left hippocampus, and right cuneus in the Tai Chi group, indicating that the Tai Chi (Bafa Wubu) intervention improved the efficiency of information transmission between these regions and other regions in the whole brain. The cingulate gyrus is an important part of the limbic system, which has rich connections with other brain regions, such as the thalamus and basal ganglia. The cingulate gyrus regulates emotion, memory, autonomic nerve function, and motor behavior. The posterior cingulate and the precuneus (located in the posterior cingulate) form a functional unit, play a key role in cognitive function (Raichle et al., 2001; Leech and Sharp, 2014). The posterior cingulate and precuneus are central to the default mode network (DMN), can be posited as a tonically active region of the brain that may continuously gather information about the world around, and within us (Raichle et al., 2001). In MCI and AD patients, connectivity between the posterior cingulate and precuneus is more likely to be disrupted. Previous studies have found that exercise can enhance the functional connectivity between the posterior cingulate and the precuneus (Chirles et al., 2017). Tai Chi Chuan is a “moving meditation” (Yao et al., 2021). It involves elements of mindfulness and meditation that constantly guide the individual to focus on himself (breathing, movement, etc.) and the surrounding environment (temperature, wind, etc.). During the Tai Chi Chuan exercise, the participants' perceptual ability is constantly enhanced, and posterior cingulate and the precuneus are frequently exercised. It may thus increase the efficiency of the two brain regions in transmitting information to other nodes. The hippocampus is a key brain region for memory formation and spatial representation. Regular physical activity has been found to be associated with increased hippocampal volume and cognitive function (Killgore et al., 2013). Tai Chi Chuan involves sequences moving memory and planning, inhibition of incorrect movements, etc. Compared with normal aerobic exercise, Tai Chi involves more memory components and frequently activates participants' memory function during exercise, which may be related to the improvement of global hippocampal efficiency. In addition, the meditative elements of Tai Chi Chuan may also induce the improvement of hippocampal efficiency. Luders and his colleagues found that individuals with long-term meditation experience had larger left and right hippocampal volumes than in controls (Luders et al., 2013). Our study also produced results that were inconsistent with the above studies. We found there is no significant improvement in hippocampal function in the brisk walking group. The study by Killgore et al. (2013) was a cross-sectional comparison of subjects with long-term exercise experience. In our study, the exercise intervention lasted for 8 weeks, and the effect of brisk walking may not have been apparent.

### Cognitive Flexibility Improved With Tai Chi Chuan Intervention

This study found that cognitive flexibility performance was significantly increased in the TCC group compared to the AE group. This finding indicates Tai Chi Chuan intervention could promote cognitive flexibility. This conclusion agrees with the findings of prior studies showing that Tai Chi Chuan participants presented with greater reductions in task-switching errors (Wu et al., 2018). In this study, Tai Chi practitioners were required to shift from one form of Bafa Wubu to the next till the end of a total of 32 forms. As previous studies have found, the benefits of cognitive flexibility may be due to the switching between different movements during Tai Chi Chuan practice (Wu et al., 2018). Interestingly, we also found that 8 weeks of brisk walking practice did not affect cognitive flexibility or brain functional network properties. Although brisk walking is a simple repetitive exercise, it is the same as Tai Chi Chuan in terms of exercise intensity, exercise frequency, and the other parameters in this study. Previous studies have found that moderate-intensity aerobic exercise can improve individual cognitive function (Guiney and Machado, 2013; Chen et al., 2014, 2016; Xiong et al., 2018), which is inconsistent with the results of this study. One reason may have to do with the subjects. There is evidence that the effects of physical activity on cognitive function are more pronounced in adolescents and older adults, while the effects are reduced in young adults (Voss et al., 2011; Hotting and Roder, 2013). The improvement of cognitive function and brain function in young adults may require movement involving more cognitive operations. Another reason may be related to the exercise intervention period. In this study, the exercise intervention lasted for 8 weeks. Our previous studies have found that 8 weeks of brisk walking can increase the volume of gray matter in the left precuneus. This indicates that brisk walking has a positive effect on brain structural plasticity, but its positive effect has not been seen to be manifested in behavior and brain functional activities. This suggests that longer intervention periods may be needed.

The Tai Chi Chuan intervention induced nodal clustering coefficient change in the left thalamus was a significant predictor of cognitive flexibility. Existing studies indicate that Tai Chi Chuan exercise improves cognitive function by improving brain functional plasticity. However, our study is the first to find that Tai Chi Chuan intervention improves cognitive flexibility by changing the topological properties of brain networks. Our study suggests that the brain function mechanism of Tai Chi Chuan to improve cognitive flexibility may be that TCC makes brain function more specialized.

In summary, the changes in global network attributes and node attributes of the TCC group reflect the improvement in brain function separation level, and brain function tends to become more specialized. Additionally, brain regions with significant changes in node attributes involve the individual's sensory perception, attention, working memory, inhibition and control, behavioral flexibility, meditation, emotion, emotional stability, decision-making, spatial memory, and other functions. In particular, the improvement of NC_*P*_ in the left thalamus predicted the improvement of cognitive flexibility. However, brisk walking has no significant effects on the topological properties of brain functional networks or cognitive flexibility. All of these findings reflect the unique advantages of Tai Chi (Bafa Wubu) in brain functional plasticity, as well as the potential to promote the healthy development of an individual's body, mind, and brain.

### Study Limitations

These results suggest a potentially more positive effect of Tai Chi Chuan on brain functional network plasticity and cognitive flexibility in comparison with general AE. However, the findings should be interpreted cautiously due to the sample size and atlas used. There is the possibility of the existence of more subtle changes in spontaneous neural activity. Larger sample sizes and longer exercise duration studies are warranted to confirm these results in future studies. The changes in other brain regions found in this study may be related to other behavioral changes caused by Tai Chi (Bafa Wubu), such as emotion, mindfulness level, and attention. The influence of Tai Chi (Bafa Wubu) on these behavioral indicators warrants further analysis. We only explored the difference between Tai Chi Chuan and general aerobic exercise on global network property and nodal property changes, and the influence of interregional functional connectivity, such as NBS, warrants further analysis.

## Conclusions

Our results demonstrated that Tai Chi Chuan was able to reshape the brain functional network and enhance functional specialization. Additionally, the enhancement of brain functional specialization by Tai Chi Chuan exercise was a predictor of higher cognitive flexibility.

## Data Availability Statement

The original contributions presented in the study are included in the article/supplementary material, further inquiries can be directed to the corresponding authors.

## Ethics Statement

The studies involving human participants were reviewed and approved by Institutional Review Board of the National Key Laboratory of Cognitive Neuroscience and Learning. The patients/participants provided their written informed consent to participate in this study.

## Author Contributions

H-cY and ST conceived the experiment and edited the manuscript. LC designed the experiment, participated in the exercise intervention, collected and analyzed the data, and wrote the manuscript. Q-qS participated in the exercise intervention, collected the data, and analyzed the data. YW and L-nZ participated in the exercise intervention and collected the data. X-jL participated in the exercise intervention. All authors reviewed the manuscript.

## Conflict of Interest

The authors declare that the research was conducted in the absence of any commercial or financial relationships that could be construed as a potential conflict of interest.
